# Enhancing Skin Cancer Diagnosis Using Swin Transformer with Hybrid Shifted Window-Based Multi-head Self-attention and SwiGLU-Based MLP

**DOI:** 10.1007/s10278-024-01140-8

**Published:** 2024-06-05

**Authors:** Ishak Pacal, Melek Alaftekin, Ferhat Devrim Zengul

**Affiliations:** 1 Department of Computer Engineering, Igdir University, 76000 Igdir, Turkey; 2 Department of Health Services Administration, The University of Alabama at Birmingham, Birmingham, AL USA; 3 Center for Integrated System, School of Engineering, The University of Alabama at Birmingham, Birmingham, AL USA; 4 Department of Biomedical Informatics and Data Science, School of Medicine, The University of Alabama, Birmingham, USA

**Keywords:** Medical image analysis, Skin cancer detection, Swin Transformer, Vision transformer, SwiGLU-based MLP

## Abstract

Skin cancer is one of the most frequently occurring cancers worldwide, and early detection is crucial for effective treatment. Dermatologists often face challenges such as heavy data demands, potential human errors, and strict time limits, which can negatively affect diagnostic outcomes. Deep learning–based diagnostic systems offer quick, accurate testing and enhanced research capabilities, providing significant support to dermatologists. In this study, we enhanced the Swin Transformer architecture by implementing the hybrid shifted window-based multi-head self-attention (HSW-MSA) in place of the conventional shifted window-based multi-head self-attention (SW-MSA). This adjustment enables the model to more efficiently process areas of skin cancer overlap, capture finer details, and manage long-range dependencies, while maintaining memory usage and computational efficiency during training. Additionally, the study replaces the standard multi-layer perceptron (MLP) in the Swin Transformer with a SwiGLU-based MLP, an upgraded version of the gated linear unit (GLU) module, to achieve higher accuracy, faster training speeds, and better parameter efficiency. The modified Swin model-base was evaluated using the publicly accessible ISIC 2019 skin dataset with eight classes and was compared against popular convolutional neural networks (CNNs) and cutting-edge vision transformer (ViT) models. In an exhaustive assessment on the unseen test dataset, the proposed Swin-Base model demonstrated exceptional performance, achieving an accuracy of 89.36%, a recall of 85.13%, a precision of 88.22%, and an F1-score of 86.65%, surpassing all previously reported research and deep learning models documented in the literature.

## Introduction

The human skin is a vital organ, constituting the primary barrier against the external environment. It acts as a barrier, protecting the human body from pathogens, foreign substances, and environmental factors. However, exposing human skin to external elements also increases the risk of developing skin diseases [1]. Cancer, which is one of the most severe threats to global health, affects millions of people worldwide. Skin cancer develops when skin cells grow abnormally. According to the 2020 Global Cancer Statistics Report issued by the International Agency for Research on Cancer (IARC), around 1.5 million fresh skin cancer instances were identified globally [2, 3]. Skin cancer incidence rates can vary significantly between countries and regions worldwide. Some areas may experience a higher prevalence of skin cancer, while others may have lower rates [4]. These disparities can be attributed to various factors, such as levels of exposure to sunlight, skin type, and genetic factors [5].

Skin cancer can develop on the skin’s surface or deeper layers. It is typically categorized into two primary forms: melanoma and non-melanoma skin cancer, encompassing basal cell carcinoma and squamous cell carcinoma. Non-melanoma skin cancer generally manifests on the superficial layers of the skin and is typically less lethal [6]. In contrast, melanoma is a variety of skin cancers originating from the unregulated proliferation of melanocytes, the cells responsible for pigment production, and it possesses a propensity to metastasize into the deeper skin layers. While constituting only about 1% of all skin cancer cases, melanoma is the primary cause of fatalities associated with skin cancer [7]. Melanoma is a highly aggressive form of cancer; however, when recognized in its early stages, it is amenable to treatment [8]. Therefore, early detection is essential for delaying the growth of malignancies like melanoma and increasing the range of treatment options. Dermatologists’ ability to diagnose and track skin lesions is crucial to early skin cancer detection [9]. The presence of skin lesions may be an indication of skin cancer, but a definitive diagnosis requires a microscopic examination of the lesion by a dermatologist [10]. Dermatologists typically use non-invasive methods such as macroscopic and dermoscopic examinations to analyze skin lesions, but they may also employ an invasive method called biopsy. A biopsy involves taking a tissue sample from a suspicious lesion and examining it under a microscope to aid in making a diagnosis. However, this method is time-consuming and challenging. Due to the potential increase in healthcare costs and the possibility of leaving scars that can have psychosocial effects on patients, biopsy is not frequently used [11]. The dermoscopic method involves using a particular dermoscopy device to obtain more detailed images of skin lesions on the skin’s surface. Compared to the macroscopic method, this technique enables a more thorough evaluation of skin lesions by evaluating factors such as color, structure, surrounding characteristics, form, and size. Dermoscopic images allow dermatologists to do a more detailed evaluation of skin lesions, facilitating early identification and the creation of effective treatment regimens [12].

In recent years, the proliferation of computer-assisted technologies, notably the widespread integration of computer-aided diagnosis (CAD) systems, has simplified the identification of skin cancer symptoms, rendering the process more efficient, cost-effective, and expeditious for healthcare practitioners [13]. The CAD technology utilizes image processing and analysis methods, deep learning, and artificial intelligence [14, 15]. These technologies have evolved into a valuable resource for aiding dermatologists in achieving accurate diagnoses, consequently alleviating their workload [8]. Deep learning has witnessed substantial recognition as an artificial intelligence technology in recent years, especially in advancing more accurate and sensitive CAD systems [16]. Deep learning constructs a model by automatically extracting features through learning from extensive datasets [17]. Learning features from numerous sample datasets enables achieving better results [18]. Deep learning has shown its effectiveness across various domains, including facial recognition, object detection, natural language processing, medical imaging, and numerous other areas [3, 15, 19–22].

Correctly classifying skin lesions is essential for prompt detection and treatment of skin cancer. The presence of diverse and irregular images in skin lesions poses a challenging process for automatic skin cancer classification. Skin cancer classification presents several challenges. Class imbalance in datasets like ISIC can bias models towards prevalent classes, leading to poor performance on rare categories. High variability between skin lesion types makes it hard for deep learning models to learn effective features. Varying image quality and lack of ethnic diversity also affect model performance. Ensuring accurate annotations is crucial to prevent misguiding the training process. Overfitting due to high dimensionality and limited labeled examples is a concern. Training complex models on large datasets requires significant computational resources. Integrating high-performing models into clinical workflows requires speed and interpretability. While robust deep learning algorithms optimized for skin cancer address many of these issues, ethical concerns and expert-dependent labeling cases remain significant matters for specialists to address. Deep learning, a subclass of machine learning, is designed to analyze data from large datasets quickly and effectively [23]. Unlike traditional machine learning methods, deep learning, with its multi-layered structures, can automatically identify patterns and relationships within data and use this information to reach relevant conclusions. Deep learning has been facilitated by its ability to handle massive datasets and the advancement of technology, particularly the parallel computing power provided by graphics processing units (GPUs), which has increased processing speed. As a result, deep learning algorithms can work with larger and more complex datasets, producing faster and more accurate results [24, 25].

Deep learning–based techniques have gained prominence in recent years as a favored option for classifying skin cancer [26–30]. The CNN architecture represents a deep learning model characterized by many trainable parameters, rendering it particularly suitable for image processing and classification tasks. Deep learning architectures offer a highly effective solution in applications such as medical image analysis, especially in challenging tasks like capturing fine-grained variables in skin cancer using dermatological lesion images [31, 32]. Medical imaging data is typically of substantial size and complex structure and suitable for application of deep learning algorithms such as CNNs and vision transformer–based architectures [33]. This study focuses on developing an innovative model based on Swin Transformer to detect skin cancer. Using the ISIC (International Skin Imaging Collaboration) 2019 dataset [34], this model aims to classify skin lesions accurately and reliably, bringing forth noteworthy contributions. Our contributions can be summarized as follows.

- Our model improves upon the Swin Transformer by introducing the innovative hybrid shifted window-based multi-head self-attention (HSW-MSA) module. This module is specifically designed to enhance the processing of overlapping skin lesions, thereby allowing for more precise capture of intricate details and long-range dependencies.
- We enhance the Swin Transformer architecture by replacing the multi-layer perceptron (MLP) with SwiGLU (switched GLU), a refined version of the MLP utilizing the gated linear unit (GLU) module. This modification leads to enhanced accuracy, accelerated training, and improved parameter efficiency. SwiGLU facilitates more effective feature extraction and representation, thereby bolstering the Swin model’s performance on skin cancer datasets.
- Furthermore, we comprehensively discuss and compare recent advancements in vision transformer–based algorithms, particularly in the context of skin cancer diagnosis. By analyzing 42 cutting-edge deep learning algorithms, we underscore the applicability and significance of cutting-edge deep learning techniques in improving diagnostic accuracy for skin cancer.

## Related Work

Deep learning methods, especially CNN-based and recently vision transformer–based architectures, have seen an increase in the number of studies in the literature related to the detection of skin lesions associated with skin cancer, classification of moles on the skin, and identification of cancer types on the skin in recent years [1, 3, 4, 6, 9, 10, 13, 14, 17, 19, 20, 22, 24, 35–44]. Although research on skin cancer diagnosis utilizes specific datasets, many studies have predominantly relied on large publicly available datasets such as ISIC (International Skin Imaging Collaboration), HAM10000, and PH2 (Public Health Dermatology Dataset) [45–47]. The scarcity of datasets available for diagnosing skin cancer, coupled with the resemblances and variations within the same class among skin lesions, can substantially influence the effectiveness of machine learning and deep learning models. Hence, the quality of the dataset used for automated skin cancer diagnosis is paramount. Gajera et al. [26] proposed a pre-trained deep CNN model to address these issues. They introduced a DenseNet-121 model with a multi-layer perceptron (MLP), achieving high accuracy rates on datasets like PH2, ISIC 2016, ISIC 2017, and HAM10000. Sedigh et al. [48] suggested a CNN to train 97 skin lesion images (50 benign and 47 malignant) from the ISIC dataset. To overcome data scarcity, they developed a generative adversarial network (GAN) to generate synthetic skin cancer images, resulting in an 18% increase in the network’s accuracy. In another study, Rafi and Shubair [49] proposed an efficient and fast-scaled 2D-CNN architecture based on EfficientNet-B7, utilizing a pre-processed extensive dataset. The performance of the proposed architecture is compared with pre-trained VGG19 and ResNet-50 models to assess its effectiveness. Nersisson et al. [50] explored the CNN-based You Only Look Once (YOLO) to detect and classify skin lesions. They fed input data through a specifically trained fully convolutional network (FCN) combining handcrafted dermoscopic image features, such as color and texture characteristics. Through this study, they achieved an accuracy of 94%, a precision of 0.85, a recall of 0.88, and a high classification accuracy with an AUC of 0.95.

Some researchers conducted preprocessing on a dataset to understand the impact of data diversity in CNN models for classifying skin lesions. Gouda et al. [51] employed ESRGAN as a preprocessing step to augment the ISIC2018 dataset. They evaluated their proposed CNN, Resnet50, InceptionV3, and Inception Resnet models and obtained comparable results with those of expert dermatologists. Nayak et al. [52] introduced a novel CNN approach based on using meta-data to address class imbalance in skin lesion data. Their proposed method demonstrated high performance in classifying skin lesions. Hosny et al. [53] presented modified Alex-net, ResNet101, and GoogleNet models, focusing on the last three layers to categorize different types of skin cancer. They demonstrated the system’s accuracy using transfer learning to overcome the issues of class imbalance and overfitting. In another research effort by Nie et al. [54], the objective was to lessen the class imbalance within the skin lesion datasets. They presented using an integrated CNN transformer model with a focal loss (FL) function for end-to-end classification on the ISIC 2018 dataset. The features obtained through the CNN architecture supported by the vision transformer were passed through a multi-layer perceptron (MLP) head for classification. The experimental analysis concluded that both the hybrid and CNN models based on FL achieved impressive results in classifying current skin lesions. Mendes and Krohling [55] proposed a new approach for classifying skin lesion clinical images, which combines features from CNN, handcrafted features, and patient clinical information. They emphasized the importance of using clinical metadata by employing a fusion architecture on the PAD-UFES-20 dataset. Gajera et al. [56] suggested a CNN model that utilizes patch-based local deep feature extraction to enhance data diversity in dermoscopy images. The effectiveness of this method was validated using the ISIC 2016 and ISIC 2017 datasets, where promising results were achieved compared to other technologies.

Akilandasowmya et al. [3] proposed a method for early skin cancer detection using ensemble classifiers and deep hidden features. They utilized SCSO-ResNet50 to distinguish hidden features and applied feature optimization with EHS to address dimensionality issues. Naive Bayes, random forest, k-NN, SVM, and linear regression were employed as ensemble classifiers for diagnosis. Naeem and Anees [24] presented DVFNet, a deep learning–based technique designed for skin cancer detection from dermoscopy images. Their approach involves preprocessing the images using anisotropic diffusion to eliminate artifacts and noise, thereby improving image quality. For feature extraction, they utilize a combination of the VGG19 architecture and histogram of oriented gradients (HOG). To handle imbalanced images within the ISIC 2019 dataset, they employ SMOTE Tomek. Zhang et al. [20] presented a methodology comprising multiple stages. Initially, input images undergo preprocessing to enhance quality and extract relevant features. These processed images are then input into a gated recurrent unit (GRU) network, selected for its proficiency in capturing sequential information. To boost the GRU Network’s effectiveness, an enhanced version of the orca predation algorithm (OPA) is utilized to refine network parameters, thereby enhancing diagnostic precision. Khan et al. [19] introduced a novel approach that utilized specialized hardware within mobile health units as data collection nodes for skin data. These data samples were then transmitted to cloud servers for processing via an innovative multi-modal information fusion framework. This framework facilitates both skin lesion segmentation and subsequent classification through two main components: segmentation and classification blocks, each incorporating performance-enhancing techniques based on information fusion principles. To segment lesions accurately, they proposed a hybrid framework that leverages the strengths of two distinct CNN architectures. In a similar vein, Ajmal et al. [14] presented a method for multiclass skin lesion classification by integrating deep learning with the fuzzy entropy slime mould algorithm. They augmented the dataset and trained models such as Inception-ResNetV2 and NasNet Mobile following fine-tuning procedures.

As indicated by the recent literature, there has been a rising fascination with employing deep learning technologies to diagnose skin cancer, driven by notable advancements in these technologies. Deep learning’s ability to process large datasets and recognize patterns has significantly impacted the diagnosis of high-risk diseases like skin cancer. Although these models used for skin cancer detection have demonstrated impressive results across various datasets, they often exhibit shortcomings in generalization, computational efficiency, detection of small lesions, scalability, and interpretability. Additionally, many proposed models have been evaluated on small datasets, and even studies using comprehensive datasets like ISIC 2019 often resort to binary splits such as train-val or train-test, which increases the risk of overfitting. This approach compromises the models’ performance on unseen test datasets, indicating a lack of generalization capability and effectiveness. Our study, however, considers the train-val-test split and focuses on performance on unseen test data, highlighting the need for a new model that addresses these deficiencies. This model utilizes hybrid shifted windows and switched GLU-based MLP for better detection of small and subtle lesions and reduces computational demands, making it more effective in clinical settings. Consequently, our proposed model not only exhibits high generalization capabilities but also implements the most advanced standards for deep learning models, offering an efficient methodology and performance.

## Method and Materials

### Dataset

**Fig. 1:**
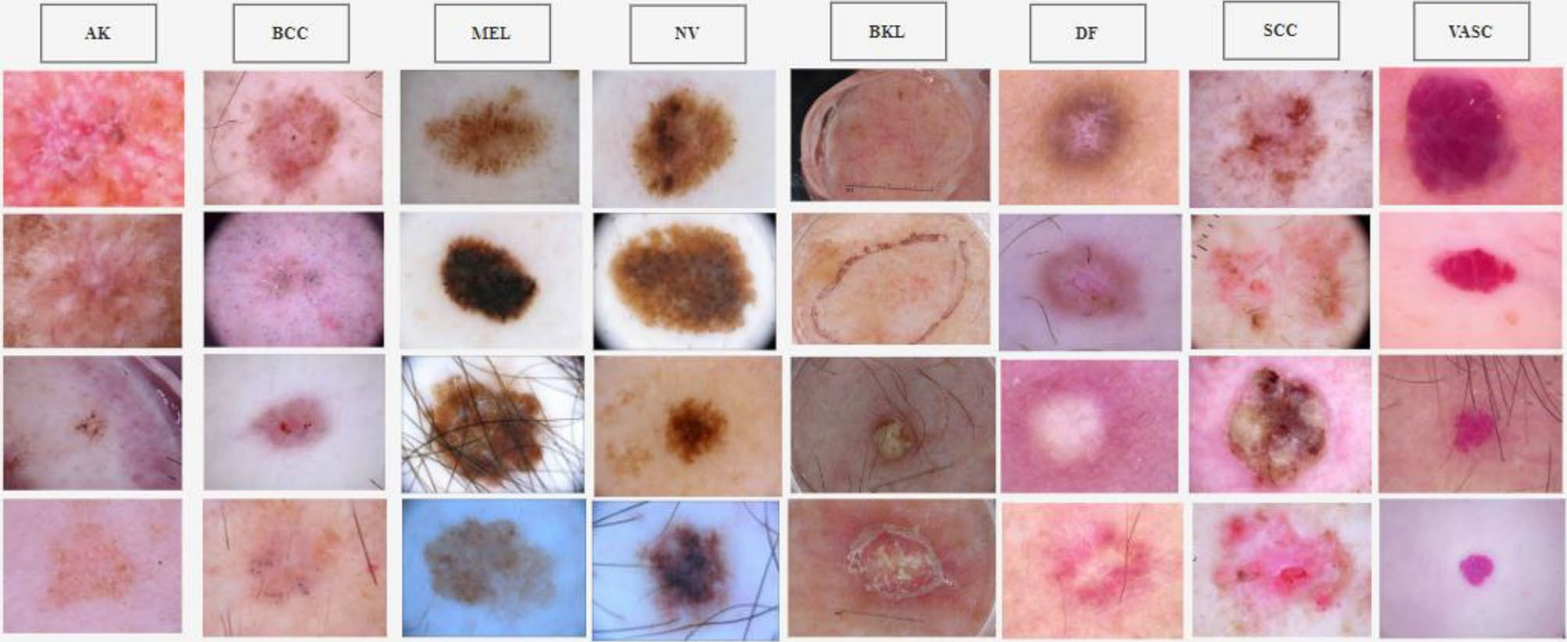
Sample images from ISIC 2019 skin lesion dataset

In this study, we employed the ISIC 2019 dataset [2], which was specifically developed for the classification of skin lesions using deep learning models. This comprehensive dataset encompasses a total of 25,331 dermatoscopic images spread across eight diagnostic categories: melanoma (MEL) with 4522 images, melanocytic nevus (NV) with 253 images, basal cell carcinoma (BCC) with 3323 images, actinic keratosis (AK) with 867 images, benign keratosis (BKL) with 2624 images, dermatofibroma (DF) with 239 images, vascular lesion (VASC) with 12,875 images, and squamous cell carcinoma (SCC) with 628 images. To enhance the model’s ability to generalize across various skin lesions, all images from each category were utilized, enriching the diversity of examples the model was trained on. However, it is important to note that the dataset predominantly consists of images from patients with lighter skin types, reflecting the demographic characteristics of the regions where the data was collected. This demographic feature is a crucial aspect that could influence the model’s performance and its generalizability to other skin types. Figure 1 displays sample images based on classes from the ISIC 2019 dataset.

### Proposed Model

Our proposed approach for effectively diagnosing skin cancer involves tailoring the Swin Transformer architecture specifically for this purpose, as it belongs to the latest vision transformer–based frameworks. The Swin Transformer, a novel architecture developed by Microsoft, introduces a hierarchical transformer whose representation is computed with shifted windows. This structure facilitates efficient model scaling and adapts more flexibly to various image sizes. By utilizing small non-overlapping local windows and shifting these windows between consecutive self-attention layers, the Swin Transformer reduces computational complexity and enhances the model’s ability to capture long-range interactions between image patches. The Swin Transformer architecture is analyzed in four stages. Initially, the input image (*H* × *W* × 3) is divided into patches, resembling ViT. Each patch, referred to as a “token,” is subjected to dimensionality compression and processed through transformer blocks that form its core structure. These patches retain their initial count and are directed to a transition block. Following this, patch merging layers organize tokens into a hierarchical format, decreasing their number while increasing the dimensions of the output. The Swin Transformer blocks preserve the resolution, repeating this sequence across multiple stages to produce different output resolutions. Initially, STBs incorporate two consecutive MSA modules: the W-MSA and the SW-MSA, each preceded by an LayerNorm (LN) layer. Following this, the architecture applies a two-layer MLP featuring a GELU non-linearity. Each of these modules reconnects to the LN layer. According to the equations presented as Eqs. 1 and 2, the MSA demonstrates quadratic computational complexity in relation to the number of tokens. However, this complexity reduces to linear when the window size *M* remains fixed, typically at 7. This design enhances the Swin Transformer’s performance, making it more efficient than the traditional Transformer model.

1 $$\Omega \left(MSA\right)={4hw C}^{2}+2 {\left(hw\right)}^{2}C$$

2 $$\Omega \left(W-MSA\right)={4hw C}^{2}+2 {M}^{2}hwC$$

In later stages of Swin Transformer blocks (STBs), a technique involving shifted window partitioning is implemented, alternating between two settings. This method uses overlapping windows to establish connections across windows while efficiently managing the calculation of non-overlapping windows. Initially, a straightforward partitioning technique is applied, dividing an 8 × 8 feature map into four 4 × 4 windows (*M* = 4). Following this, the next module shifts these windows by half their dimension, or (⌊*M*/2⌋, ⌊*M*/2⌋) pixels, modifying the initial window layout. In the Swin Transformer framework, the computation of self-attention incorporates a relative positional bias, taking into account the spatial relationships. The attention mechanism is characterized as a mapping function involving queries (*Q*), keys (*K*), values (*V*), and the resultant vectors. For each query present in the *Q* matrix, it evaluates the attention weights for associated key-value pairs to produce the final output matrix, as depicted in the Eq. 3.

3 $${Attention }\left(Q,K,V\right)={{SoftMax}}(\frac{Q{K}^{T}}{\sqrt{d}}+B)V$$

In the Swin Transformer, the matrices for queries (*Q*), keys (*K*), and values (*V*) are sized at ${R}^{{M}^{2}xd}$, where *d* is the dimension of the query/key vectors and ${M}^{2}$ indicates the total patches within a window. The model defines relative positions on each axis within the interval [− *M* + 1, *M* − 1]. It employs a relative positional bias, represented by an offset matrix $\widehat{B}$ within dimensions ${R}^{(2M-1)x(2M-1)}$. The components of the matrix B are derived from this offset matrix $\widehat{B}$.

**Fig. 2:**
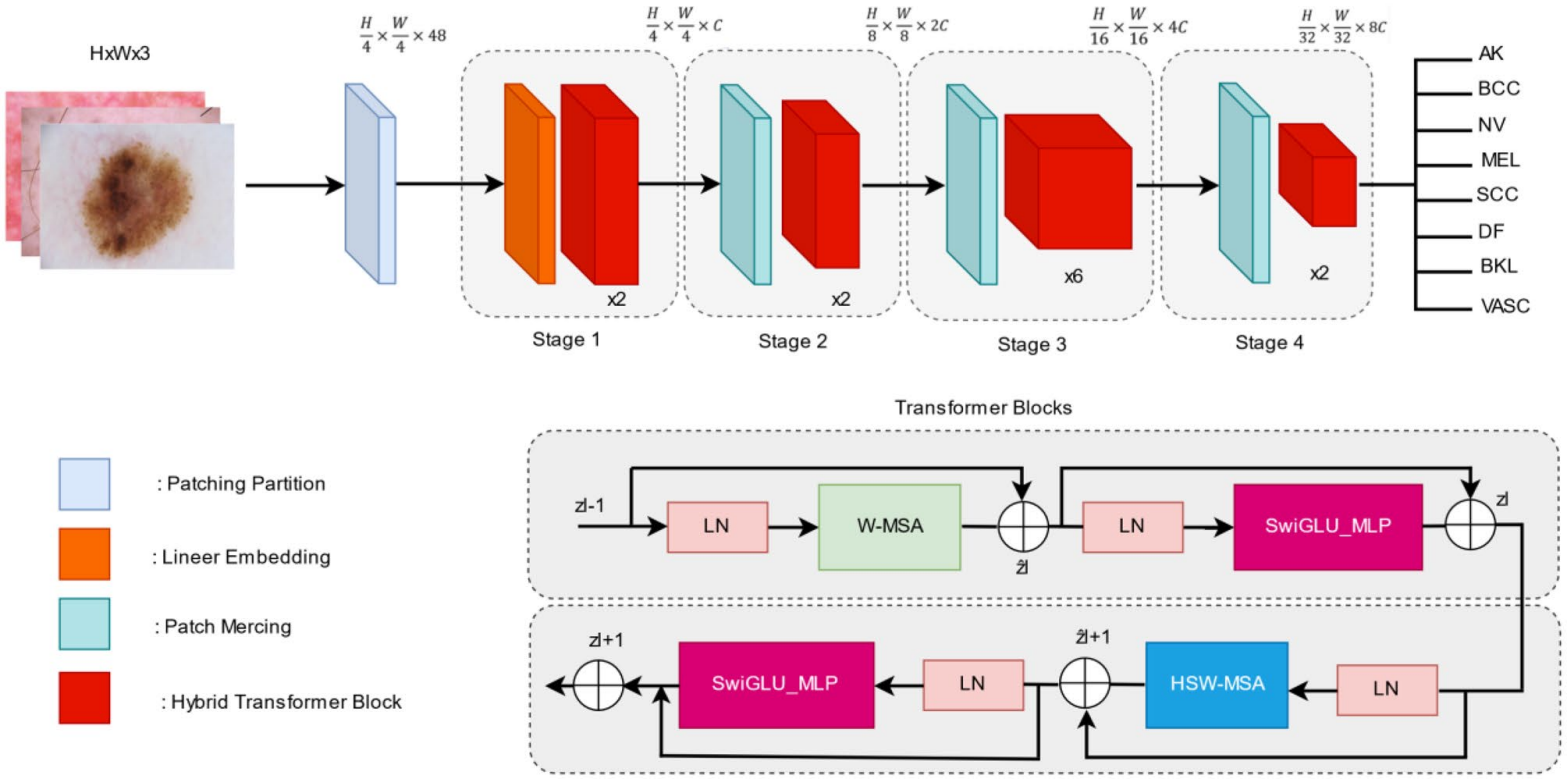
Proposed method architecture

In contrast to the pure Swin Transformer structure, we introduced fundamental improvements in the STB module and MLP module. This is because existing approaches, including models like Swin Transformer and other deep learning methods, may face challenges in achieving optimal results for similar and frequently occurring lesions, considering the unique characteristics of skin cancer images. Deep learning architectures like Swin Transformer offer specific advantages in extracting significant features through the use of attention mechanisms. However, the inherent complexity of skin cancer images can sometimes hinder even these models from obtaining satisfactory results. Particularly, the differentiation of similar lesion types and the accurate identification of common ones present limitations in models like Swin Transformer. The proposed approach aims to address challenges arising from the natural characteristics of skin cancer images and tackle crucial issues like differentiating between similar types of lesions and correctly recognizing the more prevalent ones. The architecture of the proposed method is illustrated in Fig. 2.

In the standard Swin Transformer, SW-MSA mechanism is pivotal. We enhance this by integrating hybrid shifted windows (HSW-MSA), where the window partitioning strategy is adjusted. This modification allows the model to capture both local features within each window and global context by enabling cross-window connection, vital for accurately identifying features in skin lesion images that vary subtly in texture and color. This innovation specifically addresses the challenge of overlapping skin lesion features, which are common in dermoscopic images and crucial for distinguishing between malignant and benign lesions. It is demonstrated that HSW-MSA significantly improves the model’s ability to discern overlapping and adjacent lesion boundaries, leading to more accurate classification results. On the other hand, the multilayer perceptron (MLP) layer in the Swin Transformer is traditionally a simple feed-forward network. We replace this with a SwiGLU-based MLP to introduce a gating mechanism that regulates the flow of information through the network. SwiGLU, incorporating the Swish activation function, allows for more dynamic control over feature activation, reducing the risk of overfitting on less prominent features and enhancing the model’s focus on salient features crucial for skin cancer detection. The inclusion of SwiGLU has shown to improve the depth of the feature extraction process, enabling the network to handle complex patterns in dermatoscopic images more effectively. This leads to faster convergence during training and results in a higher classification accuracy, as demonstrated by our experiments on the ISIC 2019 dataset.

#### SwiGLU-Based MLP

SwiGLU integrates the Swish activation function within its structure, yielding remarkable improvements in neural network architectures such as the Swin Transformer. The critical innovation of SwiGLU lies in its ability to separate the gating mechanism from the input processing, a feature that distinguishes it from traditional GLU implementations. This separation grants SwiGLU a unique advantage: It facilitates more intricate information flow control within the network, allowing for selective modulation of feature representations. In skin cancer detection using the Swin Transformer architecture, integrating SwiGLU has proven instrumental in achieving superior accuracy. By incorporating the Swish activation function, which exhibits smoother gradients and nonlinear behavior compared to traditional activation functions like ReLU, SwiGLU enhances the model’s capacity to capture complex patterns in skin lesion images. Furthermore, the gating mechanism inherent in SwiGLU enables the model to selectively amplify or suppress features at different hierarchical processing stages, leading to improved discrimination between malignant and benign lesions.

**Fig. 3:**
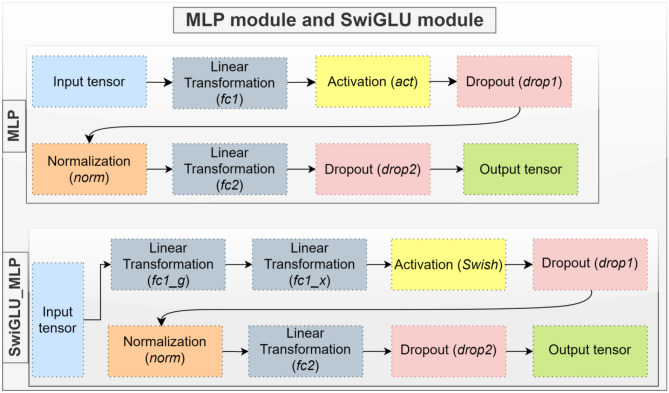
Proposed SwiGLU-based MLP module and default MLP module in Swin transformers

The effectiveness of SwiGLU in enhancing accuracy can be attributed to its unique combination of features: the smoothness of the Swish activation function and the selective gating mechanism inspired by GLU. This combination empowers the Swin Transformer to better adapt to the intricate nuances of skin lesion images, thereby improving its ability to discern subtle differences indicative of malignancy. As a result, SwiGLU emerges as a powerful tool for enhancing the performance of deep learning models in medical image analysis tasks, offering promising avenues for further research and application in clinical settings. As seen in Fig. 3, more effective training and stronger generalization capabilities were achieved by adding a SwiGLU-based MLP structure.

#### Hybrid Shifted Window-Based Multi-head Self-attention (HSW-MSA)

**Fig. 4:**
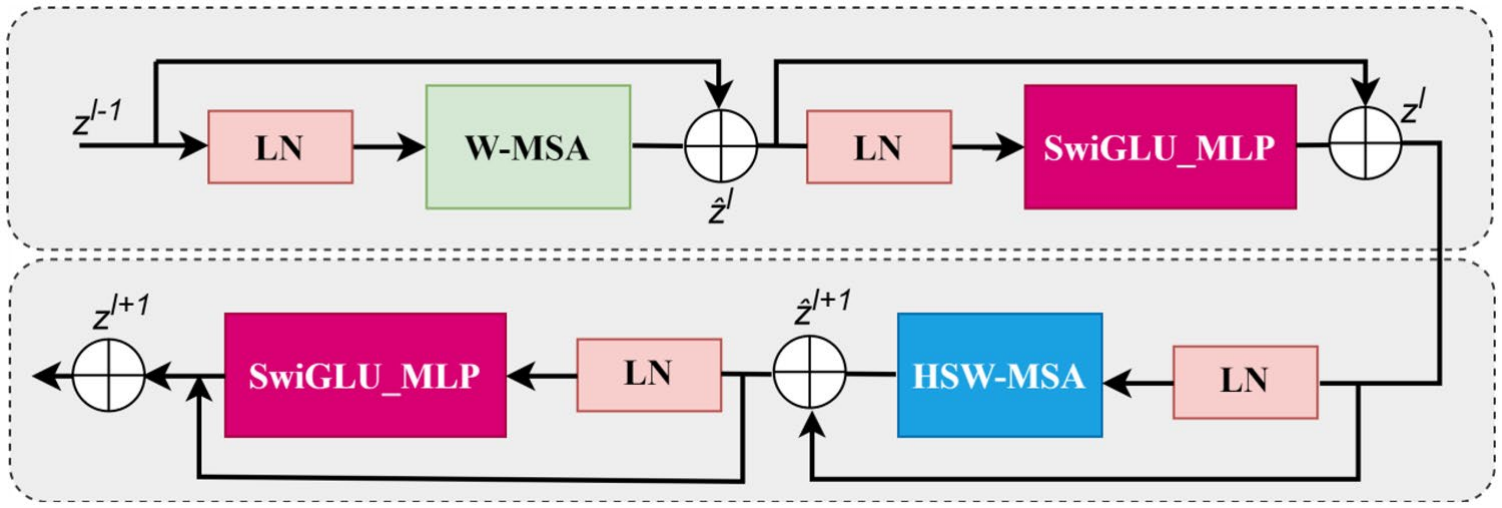
Hybrid transformer blocks

The Swin models incorporate two multi-head self-attention layers: W-MSA and SW-MSA. The enhanced proposed Swin-Base model introduces Hybrid Swin Transformer blocks using a hybrid shifted window strategy. HSW-MSA enhances traditional transformer self-attention by merging window-based and shifted techniques. This modification boosts processing efficiency and model effectiveness for large-scale data by improving detail capture and long-range dependency management, balancing computational and memory demands. This technique segments the input image and applies attention to each part, capturing relationships across patches and preserving context. The model’s hybrid self-attention module combines traditional and extended rectangular windows to handle various window sizes, enhancing flexibility and detail retention. This ability improves scale and orientation processing, increasing versatility and reducing generalization errors, potentially enhancing performance in complex tasks like skin cancer detection. The structure of these hybrid blocks is shown in Fig. 4.

The hybrid transformer blocks depicted in Fig. 4 include two distinct self-attention modules. The first is a typical window-based multi-head self-attention layer, while the second features an HSW-MSA layer. Initially, traditional shifted window-based self-attention processes the input image using a set window size, performing self-attention operations within each window to facilitate local pattern recognition. In the subsequent module, the image is segmented into horizontal and vertical stripe windows for self-attention, enhancing detail and pattern analysis across different sections. Including horizontal and vertical stripes enables the establishment of longer-range connections, thereby addressing a broader context. These three different sliding window processes enrich the multiple heads of HSW-MSA and facilitate more comprehensive visual information exchange by handling patterns at different scales. This technique is particularly useful for achieving better performance in visual processing applications. As illustrated in Fig. 4, the computation of hybrid transformer blocks is as follows:

$$\hat{z}^{l}={{W}}-{{MSA}}\left({{LN}}\left({z}^{l-1}\right)\right)+{z}^{l-1},$$

$${z}^{l}={{SwiGLU}}\_{{MPL}}\left({{LN}}\left(\hat{z}^{l}\right)\right)+\hat{z}^{l},$$

$$\hat{z}^{l+1}={{HSW}}-{{MSA}}\left({{LN}}\left({z}^{l}\right)\right)+{z}^{l},$$

4 $${z}^{l}={{SwiGLU}}\_{{MPL}} \left({{LN}}\left({\hat{z}}^{l+1}\right)\right)+ {\hat{z}}^{l+1}$$

Here, ${z}^{l}$ and ${\widehat{z}}^{l}$ represent the output features of the MLP module and HSW-MSA module, respectively, for the *l*th block.

### Transfer Learning

The sensitivity and speed of deep learning models are crucial factors in many applications and scenarios. Sensitivity refers to the model’s ability to produce accurate results, while speed determines the training and inference times [57]. Diverse methods and parameters can be utilized to improve sensitivity and optimize the processing speed of deep learning models [58]. One of the most commonly used methods is transfer learning [59]. A machine learning technique called transfer learning uses information from solving one problem to address another pertinent challenge. In transfer learning, a pre-trained model trained on a vast dataset for a particular task is employed as an initial foundation for addressing a distinct yet related task. In a significant study such as skin cancer, to enhance the performance of the proposed model, fine-tuning can be applied using information obtained from a model trained on a large dataset like ImageNet. The fine-tuning process involves adapting the general features and patterns learned by the model on a broad dataset like ImageNet to better suit the target problem. With transfer learning, the model can classify or recognize skin cancer images more rapidly and accurately using the previously learned weights.

### Data Augmentation

The size and diversity of the dataset are other crucial factors for deep learning models to perform well and prevent overfitting. Data augmentation can also be substantial in significant medical research areas like skin cancer. Obtaining a sufficient number of labeled data for skin cancer diagnosis can be challenging. Data augmentation can enhance the model’s performance even with limited labeled skin cancer data. When trained with various data augmentation techniques, the model can learn more general patterns and features, thus enhancing its ability to recognize different disease types, stages, or characteristics. Additionally, data augmentation can improve the model’s generalization capability while providing resistance against overfitting. New data samples can be generated on image data by applying operations such as rotation, scaling, mirroring, adding noise, or changing colors. These modifications reflect that skin cancer lesions can appear from different angles, in various sizes, and under different lighting conditions. Consequently, the model can learn a broader range of patterns and variations, enabling more effective classification or recognition of skin cancer images.

### Data Pre-processing

We opted to partition the dataset into training, validation, and testing datasets to enhance the study’s objectivity and assess the models’ ability to generalize effectively. Appropriately dividing the data into distinct subsets significantly impacts the training process of deep learning models. Correct partitioning enhances the model’s generalization ability and prevents issues such as overfitting or data scarcity. The partitioning of a dataset into training, validation, and test sets is a commonly employed approach in developing and evaluating machine learning models. The training set adjusts the model’s parameters, optimizes learning algorithms, and creates a general model. The validation dataset is utilized to mitigate model overfitting and assess various model architectures and combinations of hyperparameters. On the other hand, the test dataset is employed to determine the model’s performance on previously unseen data and quantify its capacity for generalization. The dataset consisting of 25,331 images was partitioned, with 70% (17,731 images) allocated for training, 15% (3800 images) designated as the validation set, and the remaining 15%, equivalent to 3800 images, reserved for the test set. Training, validation, and testing procedures for deep learning models strictly adhered to this distribution scheme across all models. The results in the tables demonstrate the real performance of each deep learning model, as they have been evaluated solely on the test data, showcasing the outcomes specific to each model. Table 1 provides the class counts for the skin cancer dataset used for training, validation, and testing purposes.

**Table 1:** The percentage of the skin cancer dataset

Class names	Total (%100)	Train (%70)	Validation (%15)	Test (%15)
Melanoma (MEL)	4522	3166	678	678
Vascular lesion (VASC)	253	177	38	38
Basal cell carcinoma (BCC)	3323	2326	498	498
Actinic keratosis (AK)	867	607	103	130
Benign keratosis (BKL)	2624	1838	393	394
Dermatofibroma (DF)	239	167	36	36
Melanocytic nevus (NV)	12,875	9013	1931	1931
Squamous cell carcinoma (SCC)	628	440	94	94
Total	25,331	17,731	3800	3800

### Experimental Design

In the context of this research, all experimental procedures were executed on a computer possessing the subsequent specifications. The operating system used was Ubuntu 22.04, the most suitable choice for deep learning platforms on Linux. The hardware configuration of this computer includes an Intel® Core™ i7-12700 K Processor, 64 GB DDR5 (5200 MHz) RAM and an NVIDIA RTX 3090 graphics card. The NVIDIA RTX 3090 graphics card consists of 10,496 CUDA cores and 328 tensor cores and utilizes 24 GB GDDR6X memory with a 384-bit memory interface. For programming, Python was used, along with PyTorch framework and NVIDIA CUDA Toolkit 11.7.

In our research, we strategically selected hyperparameters to ensure fairness, transparency, and reproducibility, while aiming for optimal balance between computational efficiency and predictive performance. For most models, the input resolution was standardized at 224 × 224 pixels, except for FlexiViT and SwinV2 which utilized 240 × 240 and 256 × 256 pixels, respectively. This standard resolution choice is based on common practices from ImageNet challenges, which has shown to be effective in balancing computational demands with performance. We trained each model for a substantial duration of 400 epochs to thoroughly learn from the dataset, using stochastic gradient descent (SGD) as the optimization algorithm with a starting learning rate of 0.01. This learning rate was chosen for its general efficacy in achieving good convergence rates in various deep learning applications.

The choice of an initial learning rate of 0.01 and a decay factor of 0.5 is justified by the need to balance the rate of convergence and the risk of diverging. Mathematically, this is modeled by *LR*_*t*_ = *LR*_*0*_ × decay^*t*/epoch length^, where *LR*_*t*_ is the learning rate at epoch* t*, *LR*_0_ is the initial learning rate, and *decay* is the decay rate per epoch. Additionally, the model weights were updated using an exponential moving average (EMA) with a decay rate of 0.9998, following the formula *W*_*t*_ = decay × *W*_*t*−1_ + (1 − decay) × *W*_new_. This ensures a stable and consistent adjustment in the weights, enhancing the model’s stability over iterations. Practical considerations were also meticulously addressed, including setting the momentum at 0.9 to help mitigate oscillations during optimization, and a minimal weight decay of 2.0e − 05 to prevent overfitting without significantly compromising the training dynamics. The training regimen included a warm-up phase where the learning rate gradually increased from a minimal 1.0e − 05 over the first five epochs, preparing the model for more aggressive learning without the initial shock of high gradient updates. Moreover, to tackle the common pitfalls of overfitting and underfitting, we divided our dataset into training, validation, and test sets, assessing the model’s generalization on unseen data strictly from the test set. Monitoring was conducted for significant improvements up to 50 epochs, beyond which training would cease if no improvement was observed, further safeguarding against overfitting and unnecessary computation. These meticulous selections and justifications of hyperparameters not only underpin the robustness of our model training process but also enhance the credibility and reproducibility of our results across different deep learning architectures, thus providing a reliable foundation for further research and application.

### Evaluation Metrics

Performance metrics are commonly used to assess the performance of deep learning algorithms and understand their generalization capabilities. These metrics assess the model during and after training on validation and test datasets. They are essential in determining whether the model faces overfitting issues, gauging the effectiveness of parameter adjustments, and gaining insights into its overall performance. Performance metrics play a pivotal role in assessing the efficacy of deep learning algorithms, offering crucial insights into their capabilities and performance. Accuracy, precision, recall, and F1-score are among the primary metrics used to evaluate models during and after training on validation and test datasets. Accuracy measures the ratio of correct predictions to the total number of predictions, providing a holistic view of model performance. Precision quantifies the model’s ability to correctly identify positive instances, while recall assesses its capacity to capture all positive instances. The F1-score balances precision and recall, offering a single metric that encapsulates both aspects of model performance. These metrics are indispensable in diagnosing overfitting, optimizing model parameters, and ensuring robust performance across various tasks in academic research and practical applications of deep learning.

## Result and Discussion

### Results for Deep Learning Models

Recent advancements in deep learning and artificial intelligence present significant potential in addressing crucial health issues such as the diagnosis and treatment of skin cancer. In this study, popular CNN-based architectures were utilized alongside most-recent vision transformer–based architectures to achieve high performance in diagnosing skin cancer. The proposed modifications were rigorously tested against traditional CNNs and other Transformer-based models using a comprehensive set of metrics including accuracy, precision, recall, and F1-score. The enhanced Swin Transformer (proposed model) consistently outperformed baseline models, validating the efficacy of the HSW-MSA and SwiGLU modifications. The results demonstrate that deep learning models serve as impressive tools for skin cancer diagnosis. The performance of the deep learning models used in the study on the ISIC 2019 test dataset is presented in Table 2. These results were obtained by following default settings and standard protocols for all model such as same default hyper-params, dataset splitting (%70 train set, %15 validation set, %15 test set) ensuring a fair comparison between them. The results in the tables demonstrate the real performance of each deep learning model, as they have been evaluated solely on the test data, showcasing the outcomes specific to each model.

**Table 2:** The experimental results of the deep learning models used in the study on the ISIC 2019 dataset

Model	Accuracy	Precision	Recall	F1-score
VGG16 [60]	0.7986	0.7983	0.6997	0.7458
ResNet50 [61]	0.7510	0.6644	0.6844	0.6743
DenseNet121 [62]	0.7836	0.7375	0.7245	0.7309
EfficientNetv2-Medium [63]	0.8273	0.7850	0.7759	0.7804
Swin-Tiny [64]	0.8236	0.7877	0.7646	0.7760
Swin-Small	0.8318	0.8083	0.7706	0.7890
Swin-Base	0.8492	0.8330	0.7931	0.8126
Swin-Large	0.8402	0.8239	0.7914	0.8073
Swinv2-Tiny-Window8 [65]	0.8278	0.8084	0.7659	0.7866
Swinv2-Tiny-Window16	0.8381	0.8036	0.8073	0.8054
Swinv2-Small-Window8	0.8371	0.8146	0.7787	0.7962
Swinv2-Small-Window16	0.8558	0.8417	0.8097	0.8254
Swinv2-Base-Window8	0.8476	0.8203	0.8042	0.8122
Swinv2-Base-Window16	0.8492	0.8263	0.8078	0.8169
Swinv2-Large-Window12-291	0.8400	0.8058	0.7992	0.8025
Swinv2-Large-Window12-256	0.8529	0.8322	0.8063	0.8190
ViT-Small-Patch16 [66]	0.8286	0.8289	0.7937	0.8109
ViT-Small-Patch32	0.7992	0.8119	0.7447	0.7768
ViT-Base-Patch16	0.8097	0.7950	0.7580	0.7761
ViT-Base-Patch32	0.8252	0.8255	0.7975	0.8113
ViT-Large-Patch16	0.8479	0.8311	0.7988	0.8146
ViT-Large-Patch32	0.8371	0.8052	0.7864	0.7957
MobileViT-small [67]	0.8426	0.8114	0.7947	0.8030
MobileViT-xsmall	0.8160	0.7750	0.7776	0.7763
MobileViT-small	0.7984	0.7522	0.7599	0.7560
MaxViT-Tiny [68]	0.8394	0.7834	0.7785	0.7809
MaxViT-Small	0.8402	0.8099	0.7904	0.8000
MaxViT-Base	0.8447	0.8236	0.7865	0.8046
MaxViT-Large	0.8489	0.8250	0.8064	0.8156
PiT-Tiny [69]	0.7905	0.7830	0.7325	0.7569
PiT-Small	0.8278	0.7977	0.7612	0.7790
PiT-Base	0.8450	0.8460	0.8084	0.8268
Swin-Tiny	0.8236	0.7877	0.7646	0.7760
Deit3-Small [70]	0.8292	0.8105	0.7670	0.7882
Deit3-Base	0.8400	0.8429	0.7717	0.8057
Deit3-Large	0.8489	0.8411	0.7942	0.8170
Flexivit-Base [71]	0.7931	0.7964	0.7405	0.7674
Flexivit-Large	0.8242	0.8009	0.7780	0.7893
GcVit-Small [72]	0.8376	0.8224	0.7823	0.8018
GcVit-Tiny	0.8307	0.8069	0.7612	0.7834
GcVit-xtiny	0.8355	0.8136	0.7792	0.7960
GcVit-xxTiny	0.8334	0.8043	0.7604	0.7817
**Proposed model**	**0.8936**	**0.8822**	**0.8513**	**0.8665**

The experimental results of various deep learning models employed in the study on the ISIC 2019 dataset, as detailed in Table 2, highlight the superior performance of the proposed model across multiple metrics. With an accuracy of 89.36%, precision of 88.22%, recall of 85.13%, and an F1-score of 86.65%, the proposed model significantly outperforms other models, including well-known architectures such as VGG16, ResNet50, and various configurations of EfficientNet and Swin Transformers. This superior performance can be attributed to the proposed model’s robust architecture which efficiently balances the detection of true positives while minimizing false negatives and false positives, a critical aspect in medical imaging where the cost of errors can be high. Specifically, the model’s high recall indicates effective sensitivity in identifying positive cases, crucial for conditions like skin cancer where early detection is vital. Furthermore, the model’s high precision suggests that it effectively limits the number of false alarms, which can reduce unnecessary anxiety and medical procedures. In contrast, other models, despite their effectiveness in certain scenarios, do not achieve the same level of balanced performance. For example, models like VGG16 and ResNet50, while historically significant in deep learning applications, show limitations in newer, more complex datasets like ISIC 2019. Advanced variants of the Swin Transformer and vision transformer (ViT) show competitive results but still fall short compared to the proposed model, particularly in recall and precision metrics. The proposed model efficiently utilizes attention mechanisms, allowing it to learn low-level features and high-level concepts successfully. Results from traditional architectures such as VGG16, ResNet50, and DenseNet121 generally show good performance but are relatively lower than newer ones. While these models achieve reasonable accuracy for the task, they lack consistent success in other metrics like Recall and F1-score. However, it is essential to note that models like VGG16 and ResNet50 can still produce good results in specific applications, especially when dealing with limited data and requiring lightweight architectures, making them valuable in such scenarios. However, it is essential not to overlook that models like VGG16 and ResNet50 can still yield good results in specific applications, particularly in cases with limited data and applications requiring lightweight architectures.

**Fig. 5:**
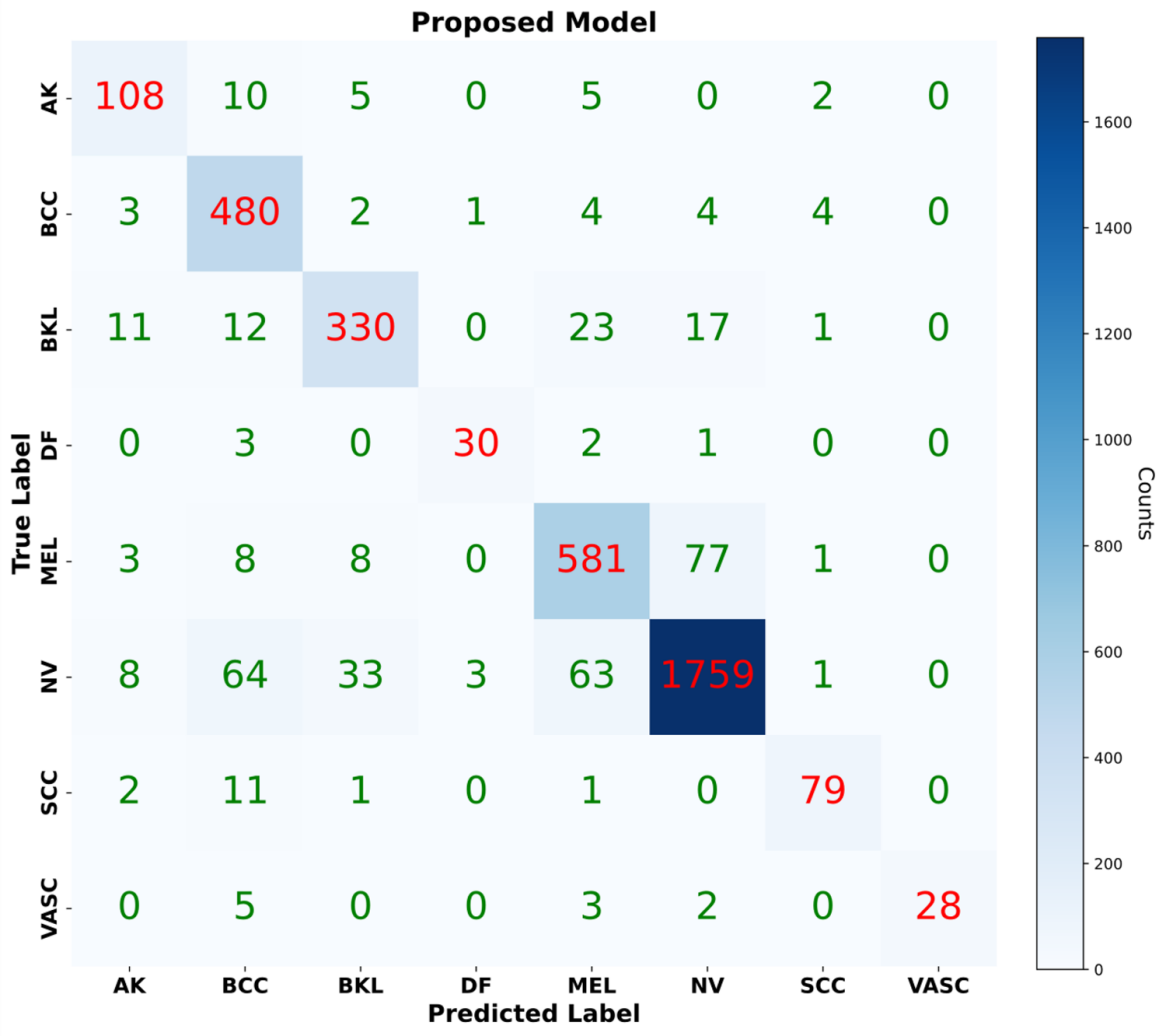
Confusion matrix of the proposed model

Among other models, Swin-Large, Swinv2-Small-Window16, Swinv2-Base-Window16, Swinv2-Large-Window12-256, ViT-large-patch-16, MaxViT-Large, Deit3-Large, and GcVit-Small have shown relatively strong performance with accuracy values ranging from 0.8402 to 0.8489. EfficientNetv2-Medium models from the Swin, ViT, and MaxViT families demonstrate better results than others. These models are built upon advanced architectures in deep learning, such as Transformers and Swin Transformers. This enables them to learn visual relationships more effectively, improving performance in various tasks. Additionally, larger models like Swinv2-Large, Swinv2-Base, ViT-large-patch-16, and MaxViT-Large outperform smaller models. However, this higher performance often comes with the trade-off of requiring more computational power and data, as larger models demand more resources. The proposed model exhibits the highest accuracy, precision, recall, and F1-score values in this study. This is attributed to the model’s ability to learn a wide range of weakly correlated visual data effectively. Furthermore, the proposed model uses a unique combination of efficient feature extraction and visual relationship learning mechanisms to enhance performance. In contrast, despite being popular architectures, models like VGG16, ResNet50, and DenseNet121 show relatively lower accuracy, precision, recall, and F1-scores. Although these models have been extensively applied in diverse computer vision assignments, their effectiveness lags behind the suggested model in this particular classification endeavor. Considering all metric values, the proposed model outperforms other models regarding higher metrics. This indicates that the model generally offers superior classification performance. Figure 5 displays the confusion matrix illustrating the class-wise performance of the proposed model. Upon examining the confusion matrix of the proposed model, it emerges as the top performer, achieving high TP and simultaneously low FP along with FN rates across most skin cancer classes.

**Fig. 6:**
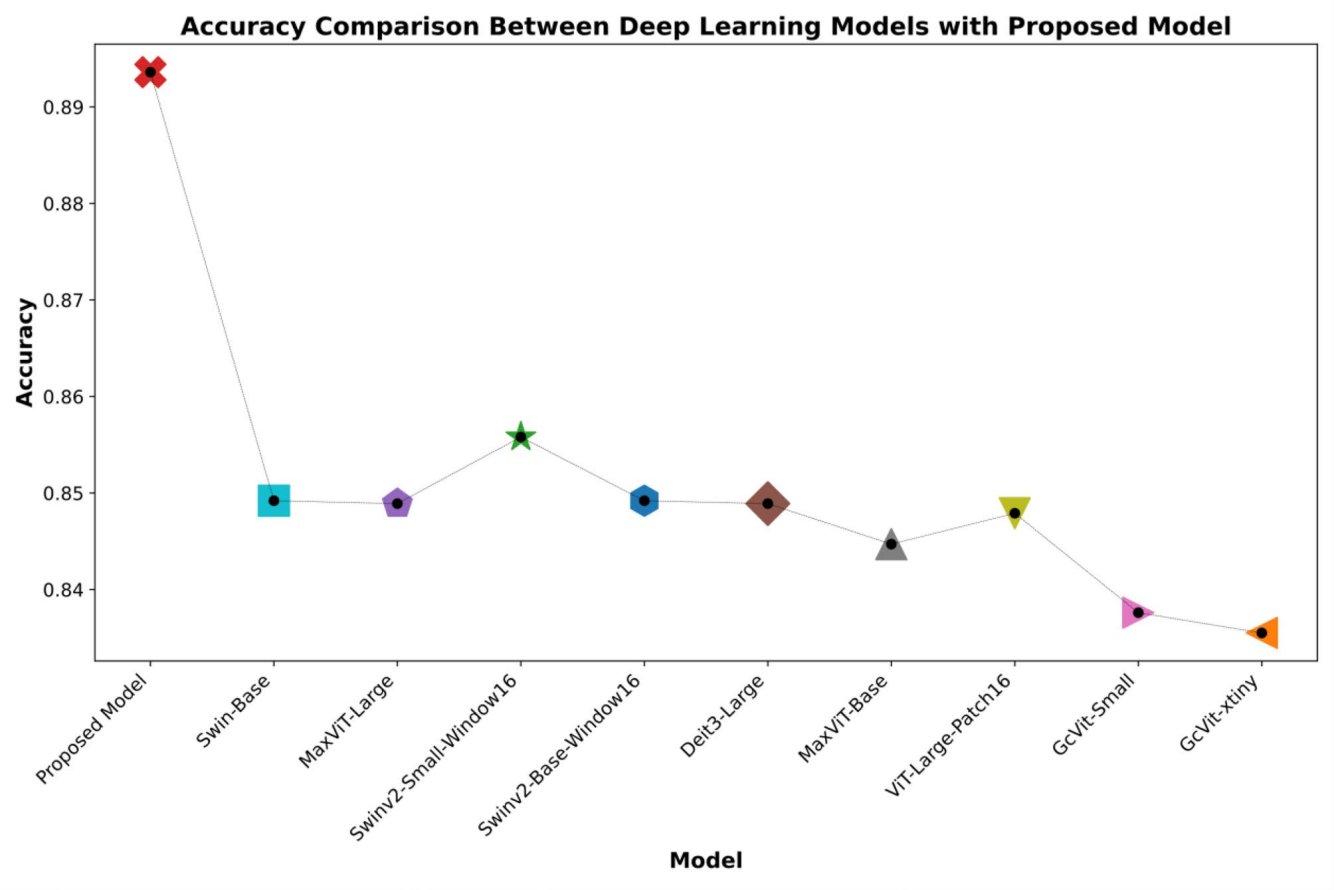
Experimental results of the proposed model alongside the top 10 deep learning models with the highest accuracy

**Fig. 7:**
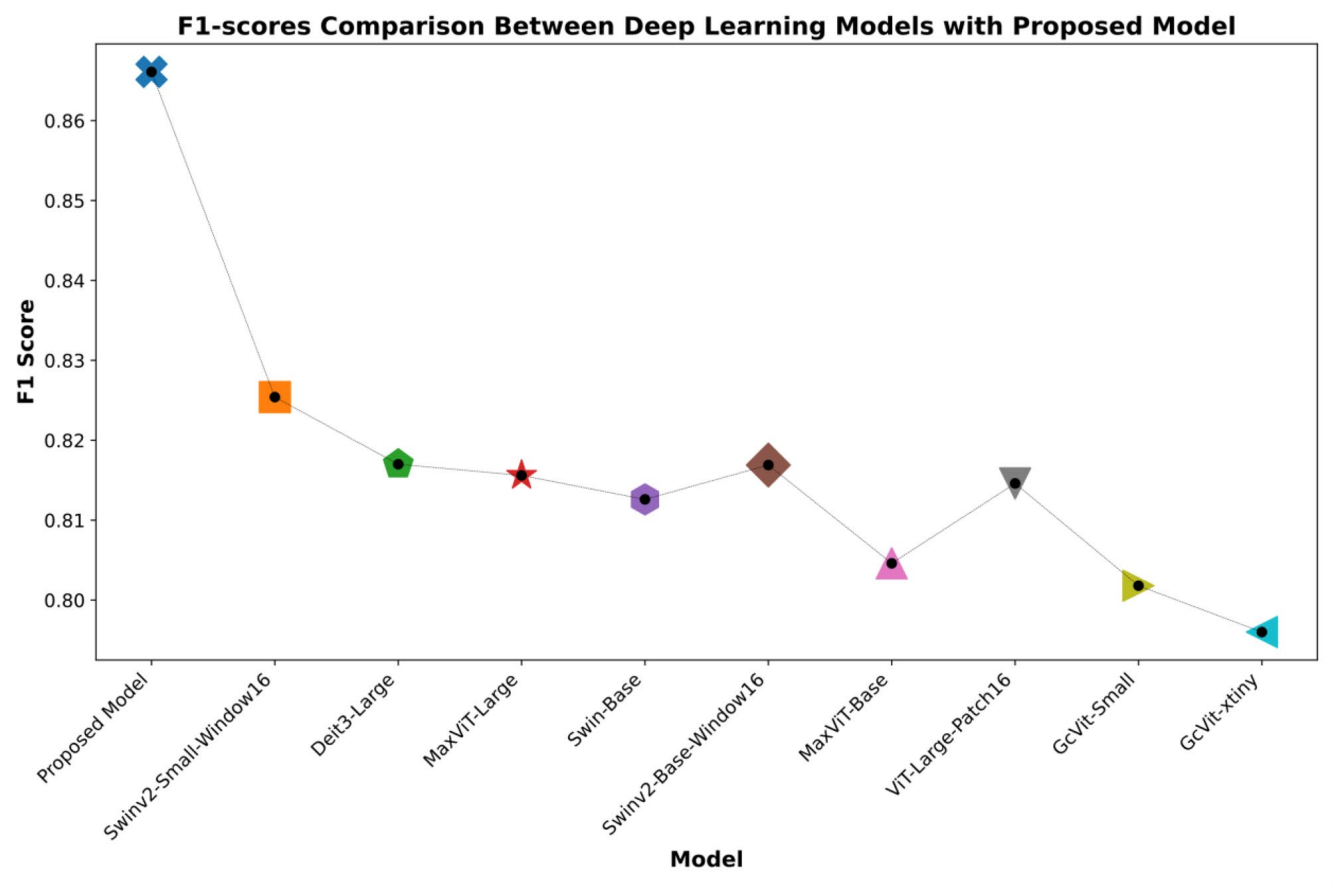
Experimental results of the proposed model alongside the top 10 deep learning models with the highest F1-score

The proposed model demonstrates its effectiveness in identifying various skin cancer types, achieving the following TP values for each class: 108 for AK, 480 for BCC, 330 for BKL, 1759 for NV, and 79 for SCC. When analyzing the FP values for each class individually, the VASC class records the lowest (0) FP value, while the highest FP value is seen in the BCC class (123 FP). Likewise, the NV class exhibits the highest FN value at 172, whereas the DF class has the lowest FN value at 6. Overall, the proposed model exhibits superior accuracy and more precise classification of diverse skin cancer types compared to other models, demonstrating its superiority across multiple metrics. In Table 2, we presented the overall results of deep learning models on the ISIC 2019 dataset, specifically the average results across the eight classes. Figures 6 and 7 display the models that achieved high performance in the experimental results. These figures specifically present a distribution graph that compares the top ten deep learning models, highlighting the proposed model with the highest accuracy and F1-scores.

Analyzing the data from Figs. 6 and 7, it is evident that the proposed model markedly surpasses other models in terms of accuracy and F1-score within the eight-class skin cancer detection task on the ISIC-2019 dataset. This model achieves an impressive accuracy of 89.36% and an F1-score of 86.61%, setting it apart from its competitors. The nearest competitor, as depicted in Fig. 6, is the Swinv2-Small-Window16, which posts an accuracy of 85.92%. Regarding the F1-score, shown in Fig. 7, the second-highest performing model is the Swinv2-Base-Window16 with an F1-score of 81.69%. The substantial lead in performance metrics of the proposed model Underscores its superiority. Enhanced by the integration of hybrid-shifted windows and the SwiGLU block, the proposed model not only demonstrates top-tier performance in detecting skin cancer but also ensures more reliable outcomes compared to other models.

### Ablation Study

In this study, we conducted an ablation analysis to assess the incremental contributions of specific architectural enhancements on the performance of our Swin Transformer models. Ablation studies are critical for understanding the efficacy of individual components within a complex model, helping to discern which elements are essential for optimal performance and which may be redundant. By systematically removing or modifying certain blocks or features, namely, the HSW-MSA and the SwiGLU-based MLP, we aimed to isolate their impacts on the model’s accuracy. This methodical approach not only clarifies the role of each component but also provides insights into the architecture’s overall design philosophy. Through this investigation, we sought to validate our hypothesis that both HSW-MSA and SwiGLU contribute positively to the model’s ability to effectively process and classify image data, thereby enhancing its predictive accuracy across various configurations. Table 3 illustrates the effects of each proposed block on Swin transformer variants and their impact on the classification of skin cancer.

**Table 3:** Outcomes of the ablation investigation regarding the impact of the proposed blocks

Block/accuracy	Swin-Tiny	Swin-Small	Swin-Base	Swin-Large
Default	0.8236	0.8318	0.8492	0.8402
HSW-MSA	0.8411	0.8497	0.8799	0.8714
SwiGLU	0.8303	0.8388	0.8645	0.8589
HSW-MSA + SwiGLU (proposed)	0.8523	0.8716	0.8936	0.8837

The ablation study detailed in Table 3 highlights the effectiveness of the HSW-MSA and SwiGLU-based MLP on the performance of Swin Transformer models when tasked with classifying skin cancer using the ISIC 2019 dataset. This dataset, known for its diverse and challenging skin lesion images, provides a rigorous testbed for evaluating the impact of architectural enhancements across different model scales: tiny, small, base, and large. Starting with the baseline (default) configurations, each model demonstrates a significant increase in accuracy with the integration of HSW-MSA and SwiGLU, individually and in combination. The addition of HSW-MSA alone markedly improves model performance, with the Swin-Base model showing a notable increase from 84.92 to 87.99% in accuracy. This suggests that HSW-MSA’s ability to enhance focus on relevant features within shifted window partitions is particularly beneficial for complex pattern recognition tasks such as those required for effective skin cancer classification. Similarly, the incorporation of SwiGLU, which facilitates improved gradient flow and nonlinear feature representation, also leads to substantial gains in accuracy. For example, the accuracy of the Swin-Large model improves from 84.02 to 85.89% with the addition of SwiGLU, indicating its effectiveness in managing the increased complexity and feature diversity in larger models. When both HSW-MSA and SwiGLU are utilized together, the models achieve the highest accuracies across all configurations. Notably, the Swin-Base model reaches an impressive accuracy of 89.36%, illustrating the synergistic effect of these enhancements in handling the ISIC 2019 dataset. This combined enhancement leads to a robust model capable of capturing a broader range of features effectively, crucial for accurately classifying the various types of skin cancer represented in the dataset. These results not only validate the individual contributions of HSW-MSA and SwiGLU but also their combined potential to substantially elevate model performance. These findings highlight the importance of component synergy in architectural design, particularly for deep learning models applied to complex tasks such as skin cancer classification from dermatoscopic images.

**Table 4:** Performance metrics (classification report) of the proposed model for the eight classes present in ISIC 2019

Class	Precision	Recall	F1-score	Number of test images
AK	0.8000	0.8308	0.8151	130
BCC	0.8094	0.9639	0.8799	498
BKL	0.8707	0.8376	0.8538	394
DF	0.8824	0.8333	0.8571	36
MEL	0.8519	0.8569	0.8544	678
NV	0.9457	0.9109	0.9280	1931
SCC	0.8977	0.8404	0.8681	94
VASC	1.0000	0.7368	0.8485	38
Macro average	0.8822	0.8513	0.8631	3799
Weighted average	0.8971	0.8937	0.8941	3799

### Class-Wise Performance of the Proposed Model

Table 4 displays an in-depth analysis of the class-specific performance of the proposed Swin-based model across the eight classes available in the ISIC 2019 dataset. This granular breakdown of metrics allows for a detailed examination of the model’s strengths and weaknesses, shedding light on its proficiency in detecting certain classes while highlighting areas where it may face challenges.

Table 4 presents the performance metrics for the proposed model across eight different skin disease categories in the ISIC 2019 dataset. Proposed model demonstrates generally high average precision (89.71%) and recall (89.37%) across various skin cancer types, with notable successes particularly in the nevus (NV) and vascular Lesions (VASC) categories. Specifically, the NV class achieved impressive results with a precision of 94.57% and recall of 91.09% across 1931 test images, while the VASC class identified lesions with 100% precision across 38 test images, though its lower recall rate of 73.68% suggests some vascular lesions were missed. Conversely, lower recall values in the dermatofibroma (DF) and again in the VASC categories (DF with 83.33% recall over 36 test images) indicate that the model occasionally overlooks certain types of lesions, particularly in rarer or less frequently sampled categories. These findings highlight areas needing improvement, particularly for medical applications where early diagnosis is crucial. Strategies such as enhancing the model with more data, refining feature extraction techniques, or altering its architecture could improve performance in weaker areas, aiming to boost diagnostic reliability overall. These strategic approaches are designed to enhance success rates in underperforming areas and elevate overall diagnostic accuracy

## Discussion

In this study, the Swin Transformer–based model (proposed model) presented offers a robust alternative for skin cancer detection, marking significant differences from existing studies in the literature. Most existing studies typically divide datasets into only training and validation (train-val) or training and testing (train-test) subsets. This approach can lead to overfitting, where models perform well on training data but fail to generalize to unseen data, diminishing their generalization ability. Although results in the literature often appear high, this could sometimes indicate overfitting, raising concerns about the reproducibility of these results across different datasets. Conversely, the training, validation, and testing (train-val-test) split adopted in this study allows for a fairer assessment of models. This tripartite division provides an opportunity to more accurately test the model’s generalization ability and enhances the reproducibility of the results. The findings from this study demonstrate that the proposed model outperforms the most advanced methods in the literature. The model has been evaluated across various performance metrics and, following extensive testing, has achieved high accuracy and precision rates in detecting various types of skin cancer.

This study’s proposed Swin Transformer–based model introduces a new approach using the ViT and specifically the Swin Transformer architecture, unlike the commonly encountered CNN-based models in skin cancer detection literature. Its unique components, HSW-MSA and SwiGLU-based MLP, have significantly improved skin cancer detection. HSW-MSA allows the model to better grasp the local and global context of images, while the SwiGLU-based MLP optimizes the training process of deep learning models, offering faster and more effective learning performance. These innovations are particularly noteworthy given the minority of studies employing ViT and Swin Transformer architectures in the literature. The reliability and generalizability of our model were tested through comprehensive comparisons with 42 different deep learning models. These comparisons demonstrate that the proposed model competes at a level with other state-of-the-art models. Moreover, detailed class-based analyses to identify the model’s strengths and weaknesses have helped overcome challenges in accurately detecting rare types of skin cancer. Additionally, the dataset was divided into train-val-test to mitigate overfitting risk, with results reported only on the unseen test set, and ablation studies, often lacking in the literature, were conducted. Ablation studies are crucial for understanding the contribution of each component to the model’s performance, helping us better understand why the model performs well or fails in certain cases. In conclusion, this study pushes the boundaries of deep learning–based approaches in skin cancer detection, offering a broader perspective compared to existing methodologies. Future research will aim to further enhance the model’s effectiveness across various skin types and ethnic backgrounds, testing its suitability for real-world clinical use.

### Limitations and Future Directions

The limitations of this study are further highlighted by the lack of diversity in skin tones represented and the existing imbalance among the classes within the ISIC 2019 dataset. The dataset predominantly features images of individuals with lighter skin tones, thus limiting the model’s accuracy across diverse ethnic backgrounds. This limitation could exacerbate disparities in performance across different ethnic groups, potentially leading to biased diagnostics. Furthermore, the class imbalance within the dataset could result in the model less effectively recognizing some classes over others, presenting a significant constraint for practical clinical applications. Although the dataset was partitioned into training, validation, and testing sets, its small scale is not ideal for deep learning models that are data-intensive. The limited size of the dataset may adversely affect the model’s generalization capabilities and hinder its adaptability to the variety of data encountered in clinical settings.

Future research should focus on enhancing the model’s efficacy under diverse skin types and conditions by utilizing larger and more varied real-world datasets. Expanding the diversity of the dataset is crucial not only for improving the model’s accuracy in identifying individuals with different skin tones but also for reducing performance variations across ethnic groups. To address data imbalance, it is essential to enrich the model with diverse and authentic data rather than relying solely on advanced data augmentation techniques. Such improvements would increase the medical accuracy and reliability of the model, offering more equitable and inclusive solutions for skin cancer diagnosis. Additionally, reducing the computational load of the model could facilitate broader clinical applications. These approaches are expected to improve the overall performance of the model, thereby supporting the wider acceptance of deep learning–based diagnostic systems in clinical environments.

## Conclusion

This research introduces a groundbreaking method aimed at tackling the complexities of diagnosing skin cancer, highlighting the critical importance of early detection for achieving optimal treatment outcomes. Employing the Swin Transformer architecture, this new approach incorporates the HSW-MSA module, enhancing the model’s ability to accurately identify overlapping cancerous regions, discern detailed features, and adeptly manage broad spatial relationships. The substitution of the traditional MLP with an innovative SwiGLU-based MLP enhances accuracy, accelerates training, and boosts parameter efficiency. The extensive testing on the ISIC 2019 skin dataset demonstrates the superior performance of the proposed Swin model, which achieved an impressive accuracy rate of 89.36%, surpassing previous leading methods in skin cancer detection and setting a new standard in the field. This study significantly advances diagnostic tools for dermatologists and researchers, illustrating the transformative impact of sophisticated deep-learning techniques on the early detection and treatment of skin cancer. It also paves the way for further advancements in medical image analysis, potentially improving patient care and outcomes in dermatology.

## Data Availability

The study data is publicly available through ISIC Challenge Dataset 2019 https://challenge.isic-archive.com/data/#2019.
